# Contributors are representative, as long as they agree: How confirmation logic overrides effort to achieve synthesis in applied health research

**DOI:** 10.1111/hex.13555

**Published:** 2022-08-11

**Authors:** Sarah E. Knowles, Pat Walkington, Jackie Flynn, Sarah Darley, Ruth Boaden, Roman Kislov

**Affiliations:** ^1^ NIHR Collaboration for Leadership in Applied Health Research and Care Greater Manchester Manchester UK; ^2^ Present address: Centre for Reviews and Dissemination University of York York UK; ^3^ Present address: School of Health Sciences, Centre for Primary Care and Health Services Research University of Manchester Manchester UK; ^4^ Present address: Faculty of Business and Law Manchester Metropolitan University, Manchester, UK and School of Health Sciences, The University of Manchester/NIHR ARC Greater Manchester Manchester UK

**Keywords:** collaborative research, coproduction, patient involvement

## Abstract

**Introduction:**

The paradox of representation in public involvement in research is well recognized, whereby public contributors are seen as either too naïve to meaningfully contribute or too knowledgeable to represent ‘the average patient’. Given the underlying assumption that expertise undermines contributions made, more expert contributors who have significant experience in research can be a primary target of criticism. We conducted a secondary analysis of a case of expert involvement and a case of lived experience, to examine how representation was discussed in each.

**Methods:**

We analysed a case of a Lived Experience Advisory Panel (LEAP) chosen for direct personal experience of a topic and a case of an expert Patient and Public Involvement (PPI) panel. Secondary analysis was of multiple qualitative data sources, including interviews with the LEAP contributors and researchers, Panel evaluation data and documentary analysis of researcher reports of Panel impacts. Analysis was undertaken collaboratively by the author team of contributors and researchers.

**Results:**

Data both from interviews with researchers and reported observations by the Panel indicated that representation was a concern for researchers in both cases. Consistent with previous research, this challenge was deployed in response to contributors requesting changes to researcher plans. However, we also observed that when contributor input could be used to support research activity, it was described unequivocally as representative of ‘the patient view’. We describe this as researchers holding a *confirmation logic*. By contrast, contributor accounts enacted a *synthesis logic*, which emphasized multiplicity of viewpoints and active dialogue. These logics are incompatible in practice, with the confirmation logic constraining the potential for the synthesis logic to be achieved.

**Conclusion:**

Researchers tend to enact a confirmation logic that seeks a monophonic patient voice to legitimize decisions. Contributors are therefore limited in their ability to realize a synthesis logic that would actively blend different types of knowledge. These different logics hold different implications regarding representation, with the synthesis logic emphasizing diversity and negotiation, as opposed to the current system in which ‘being representative' is a quality attributed to contributors by researchers.

**Patient or Public Contribution:**

Patient contributors are study coauthors, partners in analysis and reporting.

## 1 INTRODUCTION

Patient and Public Involvement (PPI), referring to the active involvement of patients, carers and members of the public in health research is increasingly recommended and even mandated by research funders. All studies receiving funding from the National Institute for Health Research (NIHR) in the United Kingdom must demonstrate (PPI) in their projects, with the Going The Extra Mile (2015) report explicitly linking PPI to principles of coproduction,^1^ which goes beyond involvement to encourage active partnership between researchers and public contributors. However, coproduction lacks a consistent definition and can be a contested activity in health research.^2^, ^3^ One common debate concerns the ‘representativeness’ of public contributors (the individuals who become involved in active or collaborative ways with researchers and the research process, as opposed to being participants in research or recipients of research findings). The ‘professionalization paradox’^4^ describes the concern that patients or members of the public who become involved in research are required to possess or gain an expert familiarity with the research process, which threatens their ability to reflect genuinely ‘lay’ viewpoints.

The coproduction literature has previously explored this paradox and critiqued the contradictory nature of the demands placed on contributors (e.g., Peter Beresford's work in Disability studies,^5^ and Diana Rose in mental health^6^, ^7^). Contributors are expected to bring individual and personal experiences yet also transcend them, and to be knowledgeable enough to have an informed opinion on research but not lose their research‐naïve public viewpoint.^8^, ^9^, ^10^, ^11^ Previous research has further argued that this characteristic of ‘being representative’ is used as a rhetorical device to reject input when contributors seek to make changes. Analyses in both health research^12^ and health commissioning^13^ therefore position the debate about ‘representativeness’ as a classic form of boundary defence employed by researchers against suggested changes, achieved by challenging the legitimacy of contributors’ knowledge as lay people.^14^

Although boundary defence may offer one understanding of the paradox of representation, there remain concerns in health research that lack of representation is a problem in PPI, which may undermine its impact or relevance. A particular target for the critique of requiring capacity to be representative are expert contributors who contribute across many studies or who have been involved in research over many years. These have been referred to as ‘super patients’ whose experience and expertise are seen as undermining their ability to represent lay perspectives.^15^ An NIHR review identified on the one hand wariness from some researchers about using experienced contributors as their expertise may dilute their experience, while other researchers felt such specialized expertise and commitment was both necessary and beneficial.^16^ Similarly in the quality improvement field, there is recognition that patient contributors may require particular skills or confidence to contribute meaningfully alongside professional stakeholders, but corresponding concern that such atypical contributors cannot reflect the concerns of wider patient populations.^17^

One possibility which is under‐explored in this debate is that both expert and lay or research naïve contributors may be required in different research activities and contexts (an option articulated by Kristina Staley^18^ in her response to one of the critiques). Research exploring the perspectives of public contributors themselves has observed that different roles can be performed by contributors within research.^19^ In a recent study of PPI across an academic health science network,^20^ Barker and colleagues proposed nine distinct roles for contributors, with one group relating to lived experience but other roles themed around citizenship or acting as an outsider. It is likely therefore that questioning whether contributors should be an expert or naïve is an over‐simplistic approach, as in practice there is a need for a diversity of roles, expertise and experience that can be performed by different contributors.

A second area of neglect in this debate is the absence of the voice of contributors themselves. Critiques of representation in PPI have largely been written by researchers—and tend to favour their perspectives as opposed to those of public contributors. While not a systematic assessment, we observe in the 16 research papers referenced so far that there was no patient involvement in nine of the studies, patient involvement described in the study but without PPI input in the paper itself in three,^13^, ^16^, ^19^ involvement in the study and paper of an academic/academics who identify as a service‐user researcher or equivalent in three^3^, ^9^, ^10^ and inclusion of a contributor coauthor alongside academic coauthors in only one.^20^ There is a need for more active collaborative analyses between researchers and contributors, exploring representation from both perspectives. Public contributors themselves are reported to be acutely aware of the paradox of representation, for example, reporting a perceived need to deliberately emphasize or underplay their expertise depending on the context, in order for their perspectives to be taken into account.^21^ This suggests that efforts to resolve the paradox or progress the debate need to work with contributors who have direct experience in navigating these tensions in practice. This paper addresses this issue by reporting a collective analysis undertaken by a team of researchers (S. E. K., S. D., R. K., R. B.) and contributors (P. W., J. F.).

We report a study conducted within the Greater Manchester Collaboration for Leadership in Applied Health Research and Care (CLAHRC) one of 13 large‐scale UK‐based applied health research partnerships funded by the NIHR in 2008–2019, which provided the opportunity to analyse descriptions of ‘expert’ and ‘naïve’ PPI roles in a collaborative context of applied health research, to consider if and how representation was discussed as these two distinct roles were enacted. Although consideration of patient involvement in summative evaluations of CLAHRCS has been lacking,^22^ evaluation of PPI activity within CLAHRCs has provided significant opportunities for critical analyses of coproduction in health research settings with learning generated that applies beyond the CLAHRC context.^23^, ^24^, ^25^ To our knowledge, however, there has not been a study of different involvement roles within a CLAHRC with consideration of how they may differ regarding perceptions of representation.

Our research questions were:

1. How is representation discussed across two cases representing expert and research‐naïve PPI?
2. How do conceptualizations of representation differ between public contributors and researchers?

## 2 METHODS

The setting for the study was an applied health research collaboration in the North West of England. For the purposes of this study, we completed a secondary analysis of qualitative data derived from two cases, which were selected from cases within a larger study exploring collaborative practice across the CLAHRC themes. The two particular cases were chosen as both involved data collected about the PPI activity and impact. Description of each case and corresponding data are presented in Table 1.

**Table 1:** Cases included in the analysis

Case	Context of work	Researcher data included in the analysis	Contributor data included in the analysis
Expert PPI Panel—‘the Panel’	Providing input across the CLAHRC portfolio of work as expert contributors, drawing on the lived experience of specific conditions when relevant but mostly providing broader input based on their generic PPI expertise	Documentary analysis of: 1. 12 months of Highlight Reports (completed by study PIs and/or theme managers every 3 months to report progress and impacts), which included reflections CLAHRC Themes (research programmes) and individual funded projects on the involvement of the panel. 2. Recruitment materials for the Panel, created by researchers.	1. Two‐hour focus group with a four‐member panel conducted to inform an internal evaluation of PPI. 2. Documentary analysis of material including ‘Strengths/Weaknesses/Opportunities/Threats’ (SWOT) feedback prepared following the evaluation.
Lived Experience Advisory Panel—‘the LEAP’	Providing input to a single CLAHRC project, where they had lived experience of the specific condition under study	Four one‐hour in‐person interviews with researchers involved in the wider study of which the LEAP was part	1. One‐hour telephone interview with one contributor
			2. One‐hour in‐person Focus group with four LEAP contributors.
			(all five members of LEAP included)

Abbreviations: CLAHRC, Collaboration for Leadership in Applied Health Research and Care; PPI, Patient and Public Involvement.

Qualitative case study research can involve a bricolage of sources and methods, as the definition of the case is emphasized over the data collection method used.^26^ Secondary analysis can similarly involve drawing on diverse sources of secondary data to produce ‘created assemblages’ that structure a comparative analysis.^27^ The assemblage is created to address questions that would not be answerable by analysis of a single project. In this study, we defined our cases according to what was being observed, meaning the discussion of public contributors, their role in research and how the discussion of being a representative was part of this, rather than the cases being defined through having equivalent data sources. Most importantly for this study, the cases were chosen because they enabled a comparison relevant to both research question 1, that is, provided an exemplar of lived experience PPI and expert contributor PPI, and research question 2, that is, provided data that enabled analytic interpretation of both contributor and researcher perspectives.

### 2.1 Analysis

Each member of the analysis team had been involved in the primary data collection in one or both cases (although the contributor coauthors had only been involved to date as participants, rather than as collaborators in study design or data collection), but all of them contributed together to the secondary analysis described here. The analysis team included P. W. and J. F. (public contributors) who were members of the Panel and S. E. K. (researcher) who conducted the Panel evaluation. R. K. (researcher) completed the interviews with the Lived Experience Advisory Panel (LEAP) researchers and S. D. (researcher) completed the interviews and focus group with the LEAP contributors. R. B. (researcher) was the director of the CLAHRC who oversaw the Panel evaluation, the highlight reports and the projects involving the LEAP.

All data were organized in NVivo. We employed both inductive and deductive thematic analysis. In the first stage, one researcher author (S. E. K.) deductively examined the data specifically to identify a discussion of representation in relation to the professionalization paradox. In the second stage, inductive analysis explored how representation was described across the two cases, how it differed across the groups involved and how this affected PPI activity and its impacts. Finally, coded extracts from the findings from stages one and two were extensively discussed among all authors, both contributors and researchers. This facilitated collective sense‐making across both groups and ensured that the resulting account incorporated—and synthesized—both contributors' and researchers' perspectives.

## 3 RESULTS

### 3.1 RQ1. How is representation discussed across two cases representing expert and research‐naïve PPI?

For this research question, we predominantly focused on the data corresponding to researcher viewpoints (interviews conducted in the LEAP case, and documentary analysis in the Panel case).

Representation was apparent as a key factor in two contrasting ways across the two cases.

First, representation was brought up explicitly in the LEAP case in the interview data from researcher participants, where it was used to justify a decision to reject contributor input. This is consistent with previous research indicating that representation is employed by researchers as a way to delegitimize contributor input when they seek instrumental change. It demonstrates however that representation critiques are not limited to ‘expert’ patient groups, but can also be employed when side‐lining those with direct lived experience.

Second, and contradicting the questioning of representation as described above, representation appeared to be unequivocally described in relation to the expert Panel as providing ‘the patient view’ in documentary data from the highlight reports. This suggests that not only was representation only questioned when refuting changes suggested by contributors but that representation is assumed when contributor feedback could be used to express support for researcher decisions.

We present illustrative data of these observations below.

#### 3.1.1 Use of representation to reject contributor input in the LEAP case

Representation was employed by two of the researchers in the LEAP case specifically to question the legitimacy of contributor input when the contributors had requested changes be made. The decision to reject the LEAP input within this narrative is justified through reference to representation:

There's the concern that … a PPI group of eight people … may or may not be representative of the…[service user] group. So a PPI group is a good starting point for consultation but if you're actually reshaping [research] then ideally you want broader [involvement]…Because you take [research] to any user group and somebody will want to make a change to it because it's something that they feel could be done differently. So the criterion we use then in terms of reviewing the changes and working with the group was is this change really, really helpful and really necessary… or is it more an individual preference that isn't really related?

Researcher Interview 1, LEAP Case

I would be looking for a range and diversity of experiences. Rather than a particular group who are working with a project who perhaps are very able and articulate and have strong feelings about certain things that can possibly overshadow some of the other aspects that could be left behind without a wider range of people in that sense. I think user groups are incredibly useful. We gain a huge amount from using them to help us understand the best way to go forward with this kind of aspect of validation… and doing all those things that enable you to get a better understanding of their perspective. But I think to have quite a small user group who have become research advisers as it were is not necessarily getting at all the experiences of [service users] in that situation.

Researcher Interview 2, LEAP Case

Both extracts illustrate that researchers referred to their assessment of how representative the LEAP may or may not be as a key consideration influencing the recjection of the requested changes. The first quote suggests that contributor input can be treated as discretionary, with a criterion of wider relevance—as opposed to ‘individual preference’—which is applied by the researcher themselves. The second quote describes contributor input as an ‘aspect of validation’ but explicitly suggests that the contributors are not representative and so the validity of their input is questionable. It was notable in the LEAP case that not all researchers held this perspective and it was a source of tension amongst the wider research team that the contributor input was being rejected in this way. However, it was also notable that this debate was not communicated back to the contributors themselves, and that the researchers' rejection of the input on this occasion was successful.

#### 3.1.2 Reference to ‘the patient perspective’ to support researcher decisions in the Panel case

In the Panel focus group, contributors reported that they suspected their input was at times dismissed by questioning whether they were representative, commenting ‘We know they call us the usual suspects’, but also reported that they were rarely informed what happened after they were consulted, and consequently did not have examples of this rejection happening. We therefore looked for evidence of how input was used in the highlight reports data. This documentary analysis of the Panel case did not find lack of representation mentioned as a challenge, but instead notably demonstrated an opposite use: researchers in the highlight reports described Panel input unequivocally as providing the patient voice in the research. This was drawn upon as validating research activities or materials. The data below show how representation is assumed, with the Panel input reflecting that of ‘a patient’:

[Panel] involvement has validated the protocol and added clarity of a how a patient would perceive being involved in the study. (CLAHRC Theme 2)

Gave a patient perspective (CLAHRC Project 1)

Made sure the language was patient friendly and information was relevant.(CLAHRC Theme 3)

Will provide the patient experience component to the final report. (CLAHRC Project 2)

It was notable that in the Panel recruitment materials, potential contributors were asked to join the panel to share their own lived experiences and personal perspectives, which is inconsistent with the focus in the Highlight reports on views that can be seen as representative of all patients. We summarize the researcher's perspective across the two cases as demonstrating a *confirmation logic*. Specifically, when contributor input provides confirmation of researcher work, it is drawn upon as representative of ‘the’ patient experience and not contested in terms of representation. When contributor input however seeks to change or debate researcher work, representation is drawn upon to dismiss input. Through this logic, only confirmation of the researcher's perspective is possible. Our analysis suggests therefore that representation is differentially applied not in regard to the expert or lived experience contributors but in regard to whether researchers receive approval or requests for change.

### 3.2 RQ2. How do conceptualizations of representation differ between public contributors and researchers?

For this research question, we brought in the data corresponding to contributor viewpoints (interviews and focus group conducted in the LEAP case, and the focus group and documentary analysis in the Panel case), enabling a comparison of the contributor accounts with the researcher accounts reported under RQ1. Representation was discussed by contributors in both cases but was conceptualized very differently from how researchers had perceived it. While the researcher's accounts suggest a preference for a monophonic ‘patient view’, the contributors themselves in both cases emphasized the need for multiplicity and diversity of viewpoints. In the LEAP case:

Respondent(R) 1: Everybody's different. We all come from different backgrounds and we're different ages.R4: And our experiences have been different as well.R2: I think I'd see ourselves representing ourselves, firstly, sort of giving our perspective from our experience. And I think that's really important, it's like everyone's got a different experience, slightly different and varied experience, and I think what we're trying to do is, at the same time then, put ourselves in not necessarily other people's shoes but trying to think, well, if that's how I feel, then I wonder how other people…

LEAP Focus Group

In the Panel case:

There is no one size fits all. Public opinion is not uniform. Any group of people will have different points of view. There is no neatness, and researchers can want to ignore lay input because it can be messy.

Panel Evaluation feedback

Both the LEAP and the Panel emphasized that an active process of negotiation through dialogue was necessary to elicit and explore these different perspectives. This is illustrated in the following quotes from the LEAP case:

My opinion of the way things should be done is going to be different to someone else's as them two versions are going to be different to a third person's; so, it's realizing that research always has to find that middle ground between everyone…. because everything's been done on a discussion basis, each and every one of us has had to compromise at some point anyway…it's put in the middle of the table and everything is discussed.

LEAP Contributor Interview

R4:We have had times when we've disagreed on certain points. And we can sit and talk about it, and see each other's perspectives on it.R1:And debate it, yeah.R4:And then come to a happy medium. That we know is going to help the people we're trying to help.

LEAP Focus Group

The Panel similarly emphasized the need for dialogue and interaction. They specifically warned against the idea of a single contributor being ‘the PPI’ on a project, as this would prevent the necessary interaction from happening. Collectively meaningful accounts were considered to be produced through interaction between contributors, rather than an individual contributor being able to represent a collective opinion. The multiplicity of perspectives and resolution through interaction with other contributors were therefore emphasized in both cases.It was also evident across both cases that contributors did not see themselves as only offering or representing ‘a patient view' but drew on different roles they occupied, for example, in professional or voluntary roles in both health and related sectors, again contributing to a multiplicity of perspectives and experiences:

I think [other contributor's] experience and knowledge of [Charity Partner] really has helped inform the team.…you know, those stumbling blocks hopefully have been reduced because having that sort of almost like inside knowledge.We're used to working with the NHS, so we sort of know our way around it …so we bring that kind of another layer of experience to the issues. it's about how you, as a person, sort of bring your life experience.

LEAP Focus Group

Some of us worked with universities and we've worked with governors and doctors and all sorts. We know what big organisations are like and what it takes to get things get done.

Panel Evaluation Feedback

While both the LEAP and Panel described the need to explore different perspectives amongst contributors, they also discussed the need for interaction between contributors and researchers. The Panel descriptions of their role focused on this as necessary interaction between different experiences and ways of knowing. The panel described themselves as being ‘intermediaries between academics and the public’ and used the language of working across or between spaces, sometimes acting as ‘translators’ between the groups: ‘We relate [what academics say] back to real lives’. Bringing this experience into combination with researcher knowledge was seen as key to their role: asked to define what they considered effective involvement, the panel described ‘the melding of two perspectives’ referring to research and lived experience perspectives being brought together. They further discussed how this interaction was dependent on researchers being open to this different knowledge:

Researchers who are open minded are key to PPI not being a tick boxWhen saying something emotive is as valuable to them as if you gave them a formula.

Panel Evaluation Feedback

The LEAP also positively described having this interactive relationship with some of the researchers on the project:

it feels like a partnership… it's actually there's been a process and a long‐term process where there's a relationship of trust, a relationship of understanding. And if we haven't understood, then we've asked the question. Or if [the researchers] haven't, they'd go and check it out again, just to make sure.

LEAP Focus Group

However, the LEAP notably was unaware of the debate about their representativeness that was articulated by other researchers on the project, and it was unclear if the researchers holding these views had communicated this rationale to either the LEAP themselves or the research team members facilitating the LEAP. Similarly, the Panel expressed frustration that they were not informed if and how their suggestions had been taken forward.

We don't know if people are doing other PPI without us or not doing it…We're not included in the reports and we hear second hand if at all.

Panel Feedback

We summarize the contributor perspective across the two cases as demonstrating a synthesis logic. This reflects first, that both LEAP and Panel perceived the goal of their contribution as being to achieve a synthesis across different viewpoints both within the groups of public contributors and with researchers (including bringing in their own diverse experiences in different roles), and second that they viewed this goal as being achieved through active synthesis, with interaction, dialogue and negotiation between public contributors' and researchers' viewpoints.

### 3.3 Analytic comparison of the two logics

It is notable in the data that synthesis with researchers is aspirational for the contributors, but not fully realized in practice. This can be seen as due to the more powerful group in this case (the researchers) being able to enact the confirmation logic, which our collaborative analysis group observed to be in opposition to the synthesis logic in three key ways:

1. While the synthesis logic encourages interaction and blending of different perspectives, the confirmation logic seeks validation of existing perspectives.
2. The synthesis logic emphasizes polyphony, with contributors themselves bringing multiple different experiences, while the confirmation logic seeks a monophonic ‘patient view’.
3. The synthesis logic emphasizes interaction and negotiation, while the confirmation logic operates as a discretionary decision made by researchers about contributor input without accountability or transparency back to those contributors.

Consequently, we conclude that the synthesis logic is currently predominantly aspirational and is inhibited in practice in cases where the confirmation logic is enforced.

## 4 DISCUSSION

Consistent with previous research, we found that representation is used by researchers as a means to defend against changes initiated by contributors. We further demonstrate that this is not limited to expert contributors but is applied to contributors with specific lived experience. This suggests that the relative expertise and experience of the contributor are secondary to whether the contributor is requesting changes be made. Our findings are therefore consistent with studies suggesting that challenges to legitimacy are most often encountered as a form of defence against instrumental impacts of PPI.^12^ Problematically, contributors are representative as long as they agree.In this paper, we have expanded further on this through the analytical observation of the confirmation logic, whereby the representation of a monophonic patient perspective is assumed when it can be used to confirm—justify or support—existing researcher decisions. We acknowledge that our analysis is based on only two cases and that our conclusion of a confirmation logic particularly is based on secondary documentary data reported for other purposes rather than being articulated in direct interviews. We suggest however that researchers in interviews may not have wished to express this logic so openly, and therefore the secondary analysis is a strength of the study. The confirmation logic also has support in other literature on patient involvement. For example, an evaluation of the impacts of James Lind Alliance Priority Setting Partnerships found that ‘researchers tended to use a Top 10 priority to strengthen the case for a study they already planned to do’, finding no evidence that an existing research topic was changed to accommodate a new priority.^28^ In the service improvement context, similarly it was reported that ‘involvement was used instrumentally by programme leaders to gain support for change the case for which had already been made, and for service models already developed’.^29^ At a systemic level, Montenegro and Cornish have argued that the role of user groups in mental health reform in Chile was driven, and later undermined, by the use of user input for legitimation.^30^ We suggest therefore that our proposed confirmation logic has validity beyond the present study and the specific CLAHRC setting, and offers a succinct way of conceptualizing this common observation.The key analytic contribution of this paper has been to contrast this conformation logic with the synthesis logic held by contributors themselves. We observe the following implications.

### 4.1 Comparing synthesis and confirmation logics in involvement 1: Monophonic versus polyphonic accounts of patient perspectives

First, there is an expressed contradiction between the researcher's preference for a monophonic patient view and the plurality of voices that are considered essential by contributors. The tendency to frame PPI as providing a singular ‘patient perspective’ has been reported previously by Rowland and colleagues,^31^ with the recommendation that clarity is needed about what ‘patient voice' is being represented and how, for example, referring to democratic, statistical or symbolic representation, which each have different requirements in terms of who is involved in research and how.^32^ If it is indeed a consensus opinion being sought, then researchers would need to adopt approaches that deliberately define and seek views of a representative group. There are established participatory methods, which deliberately seek to engage representative cohorts, for example, citizen juries.^33^ We observed in the documentary analysis however there is currently an inconsistency in how contributors were recruited (emphasizing personal experience) and how their input was then framed in reporting (representing collective opinion). Future research should aim to provide a clearer articulation of the purpose of the involvement and the criteria on which representation is to be judged. Alternatively, the polyphonic contributions described by contributors themselves may be considered more appropriate or valuable, depending on the research context. To achieve this multiplicity of viewpoints, more focus is likely needed on supporting diverse contributors to access involvement opportunities, and on providing inclusive spaces, which can effectively elicit and explore such differences.We note that in both of the cases, the onus is on researchers themselves to understand which approach is required and to make efforts to support this, positioning researchers themselves as responsible for transparently articulating what they assume contributors offer.^34^ This may be a positive direction to take, given that to date the emphasis appears to be on how contributors themselves handle the paradox, including through rhetorical use of the collective voice^35^ and negotiation of their ‘symbolic capital’ as patients.^21^ We suggest that instead researchers themselves could be considered to hold a responsibility not to place paradoxical demands on contributors. This is not however solely the responsibility of individual researchers but should be understood as operating within a wider research context that limits opportunities for flexibility and change and incentivizes an extractive approach to involvement.^36^ Within applied health research, Papoulias and Callard identified how both organizational activities and researcher behaviours, such as a focus on deliverables, create spatial and temporal logics that constrain the potential for involvement.^37^ It is notable that the confirmation logic in this study was evident via the Highlight Reporting, a managerial reporting mechanism that may have inadvertently encouraged unproblematic reporting of PPI impacts. Efforts to challenge the confirmation logic therefore should consider both organizational and individual motivations.

### 4.2 Comparing synthesis and confirmation logics in involvement 2: The need for transparency and negotiation

Second, there is a notable contrast between contributors' desire to actively negotiate knowledge, and the current system whereby researchers choose whether to accept or reject input, but without exploring the reasons for this with contributors directly. While some researchers may consider themselves to be protecting contributors from difficult discussions, we note this is a paternalistic attitude that may in effect serve to protect researchers from difficult conversations with contributors. Coproduction evaluations have argued that exploring tension in the process is both necessary and valuable.^38^, ^39^ This may be particularly necessary for applied health research settings, where a lack of awareness of or resistance to involvement can significantly limit the potential for impact.^40^ Indeed, definitions of impact themselves are likely to vary depending on the logics held around public involvement, as demonstrated in health service improvement by Greer and colleagues,^41^ and these therefore need to be surfaced and understood.Frameworks for involvement that acknowledge the different roles, processes and impacts that can be anticipated could be usefully employed to support this (e.g., Oliver et al.^42^ and Harris et al.^43^) Poland et al.^44^ adopted a critical case analysis approach to make visible the challenges and conflicts of embedding PPI in a health research programme, to explicitly discuss how different ways of knowing were managed and reconciled, observing that such work is rarely reported. Irrespective of value and impact, it can also be argued that it is unethical for current systems to fail to transparently report decisions back to contributors and that an accountability mechanism should be introduced, which ensure that researchers communicate their decisions back to contributors themselves, which should be followed by facilitating a meaningful dialogue between the two groups.The contributor co‐authors in our team reflected on how hearing at a later time or second hand that their input had been disregarded could severely damage their trust and willingness to engage in the future. By contrast, experiences of the meaningful debate were welcomed and could enhance relationships. One coauthor comments: ‘Over all the projects I have been involved in I have very much appreciated active engagement and debate amongst both contributors and researchers, and have developed an admiration for researchers who are willing to take the risk of truly listening to the lay participants, and have the courage to reflect that input in their work’.

### 4.3 Comparing synthesis and confirmation logics in involvement 3: Exploring how synthesis occurs

Finally, the synthesis logic that we describe is worthy of further investigation, particularly as this logic is consistent with current understandings of and recommendations for the production and coproduction of knowledge in health research (referring to ‘mode 2’ approaches, which emphasize the need for blending of different ways of knowing to inform health research^45^). In particular, it will be valuable in the future to better understand the nature of synthesis and its processes and impacts, particularly considering Bakhtin's idea of polyphony, which has been expressed in previous studies of involvement^11^, ^24^ and may particularly align with the synthesis logic identified in this study. In contrast to Barker et al., who suggested that lived experience may ‘lack relevance’ in relation to particular roles, the synthesis logic instead emphasizes the different kinds of lived experience that contributors bring—which can include lived experience of involvement in the research itself—and crucially emphasized interaction as a mechanism for drawing in different experiences in relevant ways.There are several possible ways in which the synthesis activity might be understood to contribute to research, for example, as a form of knowledge brokering^17^ or boundary‐spanning activity (Croft and Currie^46^ describe the untapped ‘co‐ordination capability’ of patients and Martin^8^ describes contributors' mediating role). It could also be conceived of as a type of interactional expertise. [Boivin et al.^35^ describe the need for a ‘contributory public expertise’ (p. 345), which facilitates the hybridization of knowledge, suggesting that patient involvement could be conceptualized as a particular form of interactional expertise within the ‘third wave’ of knowledge studies^47^]. We therefore encourage researchers to continue to explore this synthesis activity with reference to knowledge management literature. The contributor co‐authors on this paper suggested that such conceptualizations may be especially preferable as they position contributors as having relevant and necessary expertise to inform research, as opposed to them dismissed as ‘being professionalized’ (which is something done to them, as passive recipients, rather than recognizing they have an active and indeed highly skilled role in negotiating knowledge). Alternatively, it may be valuable to draw on culturally different ways of knowing to offer a new perspective on the apparent challenge of representation. In an NIHR Race Equality Public Action Group Thematic Analysis^48^ the concept of ubuntu or ‘I am because we are’, was articulated by contributors of Black African heritage. This concept may offer a way to reconcile what may be a Eurocentric binary between individual experience and collective representation through acknowledging these as interdependent.^49^, ^50^

### 4.4 Reframing the call for diversity in patient involvement

It should be understood that a focus on synthesis in the way we describe does not negate consideration of whose contributions are included and who may be excluded. This consideration was apparent in the contributors' discussion of the need for diversity of experience, and diversity, rather than representation, may be a productive focus for future work. Across different fields, there is consensus that coproduction efforts should explicitly attend to the need to include different ways of knowing and addressing who has power and legitimacy in these processes.^51^ Rather than only questioning whether ‘the usual suspects’ of white, professional, educated contributors can represent other views, we should also question how and why people who do not match that profile have been excluded to date.

### 4.5 Strengths and limitations

The data sources were retrospectively analysed, and we are drawing conclusions based on this secondary analysis rather than having explored this actively with participants. We acknowledge a lack of comparative data, particularly in the panel case where researchers were not directly interviewed. However, drawing on diverse forms of available data provided a novel insight into how PPI input was being described in researcher reports. It is not uncommon for case study analysis to draw across diverse naturalistic sources, and it is important to recognize that we do not suggest a cumulative data set was achieved (meaning the data is automatically comparable and can be combined), but rather we judged we had sufficient data to enable a meaningful analytic conversation across the cases that were chosen relevant to the two research questions.^52^ While we were a partnership in relation to data analysis and writing this paper (and agree with calls for greater clarity in reporting patient authorship^53^), the contributors were not involved before this in design or data collection. Researchers should be mindful that there remains a gap regarding studies that involve contributors fully and throughout the research process, from conceptualization to reporting.We were prompted to acknowledge, by an anonymous contributor reviewer, our choice of language, for example, around descriptions of ‘logics’, and whether this is accessible to lay audiences. This is an important consideration because it can be seen as another layer of exclusion that prevents contributors themselves from engaging with debates, meaning discussion is about them, without them. We explored this as a team and discussed our decision to adopt language and terminology that would be persuasive to our target audience, which in the case of this research, we felt to be the research community. The contributor co‐authors described this as a deliberate compromise, that ‘we cede the battle [around using more accessible language] but we may win the war, by converting researchers to appreciate our point of view’. Although our own group found this to be acceptable in this instance, it is an important area for future discussion, and finding mutually acceptable and valuable ways to express findings is yet another area requiring open discussion and negotiation. We also acknowledge this approach is not without risk, as we may inadvertently contribute to the privileging of knowledge that is expressed according to academic norms. We would in fact encourage researchers to recognize that sophisticated analysis of involvement concepts such as representation is often produced outside academic channels by contributors themselves (see e.g., ‘The Rep Trap’ by David Gilbert,^54^ which astutely and critically addresses the challenge of being deemed a ‘patient representative’).

## 5 CONCLUSION

We began the study with the expectation that the expert Panel may be subject to challenge over‐representation in a way the lived‐experience LEAP was not. Our findings show that relative expertise is not the deciding factor in whether representation is critiqued or not. Instead, the driver behind this appears to be the confirmation logic, whereby representation is assumed or revoked by researchers based on whether their own decisions are supported or critiqued. This demonstrates that despite ostensible commitments to equal partnership, researchers continue to hold power over decision‐making in research involvement, limiting the potential for contributors to have influence. We observed that contributors, by comparison, hold a synthesis logic, and made suggestions for how this synthesis process can be better understood in the future. We note however that such effort to understand this logic will be irrelevant, if, in practice, it cannot be enacted. Surfacing tensions in approaches to coproduction is essential to move beyond misleading debates about representation. Involving contributors themselves in these debates is both a necessity for making progress and, we suggest, an ethical responsibility.

## AUTHOR CONTRIBUTIONS

All authors were involved in conceptualizing the study. Data was collected by Roman Kislov, Sarah Darley and Sarah E. Knowles. All authors contributed to the data analysis. Sarah E. Knowles prepared the first draft of the paper. All authors commented on and approved the final submission.

## CONFLICT OF INTEREST

The authors declare no conflict of interest.

## ETHICS STATEMENT

Data collected regarding the LEAP was part of a study that received ethical approval from Alliance Manchester Business School (reference AMBS/16/03). The Panel evaluation data was Patient and Public Involvement activity and did not require committee ethical approval in line with UK guidance. Contributors gave permission for their anonymized feedback to be used in public reports, including academic publications.

## Data Availability

The data that support the findings of this study are available on request from the corresponding author. The data are not publicly available due to privacy or ethical restrictions.
